# Playfulness and New Technologies in Hand Therapy for Children With Cerebral Palsy: Scoping Review

**DOI:** 10.2196/44904

**Published:** 2023-10-16

**Authors:** Tamara Veronica Pinos Cisneros, Annette Brons, Ben Kröse, Ben Schouten, Geke Ludden

**Affiliations:** 1 Digital Life Centre Amsterdam University of Applied Sciences Amsterdam Netherlands; 2 Interaction Design University of Twente Enschede Netherlands; 3 Play and Civic Media Amsterdam University of Applied Sciences Amsterdam Netherlands

**Keywords:** technology, cerebral palsy, play, children, hand therapy

## Abstract

**Background:**

Innovative technologies such as game consoles and smart toys used with games or playful approaches have proven to be successful and attractive in providing effective and motivating hand therapy for children with cerebral palsy (CP). Thus, there is an increased interest in designing and implementing interventions that can improve the well-being of these children. However, to understand how and why these interventions are motivating children, we need a better understanding of the playful elements of technology-supported hand therapy.

**Objective:**

This scoping review aims to identify the playful elements and the innovative technologies currently used in hand therapy for children with CP.

**Methods:**

We included studies that design or evaluate interventions for children with CP that use innovative technologies with game or play strategies. Data were extracted and analyzed based on the type of technology, description of the system, and playful elements according to the Lenses of Play, a play design toolkit. A total of 31 studies were included in the analysis.

**Results:**

Overall, 54 papers were included in the analysis. The results showed high use of consumer technologies in hand therapy for children with CP. Although several studies have used a combination of consumer technologies with therapeutic-specific technologies, only a few studies focused on the exclusive use of therapeutic-specific technologies. To analyze the playfulness of these interventions that make use of innovative technologies, we focused our review on 3 lenses of play: *Open-ended Play*, where it was found that the characteristics of ludus, such as a structured form of play and defined goals and rules, were the most common, whereas strategies that relate to paidia were less common. The most commonly used *Forms of Play* were physical or active form and games with rules. Finally, the most popular *Playful experiences* were control, challenge, and competition.

**Conclusions:**

The inventory and analysis of innovative technology and playful elements provided in this study can be a starting point for new developments of fun and engaging tools to assist hand therapy for children with CP.

## Introduction

### Background

Children with hand motor skills deficiencies face challenges daily. They may have difficulties with daily activities such as eating, getting dressed, or socializing with friends and families. Physical and occupational therapies can ameliorate the motor skills of these children [1]. One group with hand motor skills deficiencies is children with cerebral palsy (CP). Despite therapists’ efforts, the interventions available for this group are repetitive and thus perceived as demotivating [2]. Providing therapy via a motivating activity positively impacts improving motor skills, as patients are more willing to take part and adhere to the treatment. Therefore, rehabilitation researchers and therapists constantly look for ways to innovate and improve existing therapies.

Challenges of current therapy at home include lacking the means for personalization, monitoring of progress of the exercises, and high cost of devices. Children with CP present with a diverse degree of motor function, and no 2 children will be affected in the same way. Therapists therefore adjust the exercises according to each child; however, this type of personalization is challenging if the therapy is to be performed at home. In addition, therapists need to provide tailored and timely feedback for the child to sustain motivation and increase adherence when performing therapy at home. Trying to provide this help at home can increase the workload and pressure on the therapists and caregivers.

New technologies, such as the E-link, the HandTutor, or the surface electromyography, present advantages in hand rehabilitation such as data analysis, customization, feedback, and adaptability to the home environment [3]; however, their high cost means that families cannot make use of them easily. Two known approaches to increase motivation are the use of new technologies and play. The benefits of play in the development of children have been widely studied, showing that the motivational nature of play encourages children to participate and learn. Thus, one of the approaches that therapists have successfully used in rehabilitation centers is to perform exercises through play [4]. One successful example of play-based therapy is Pirate Therapy [5], which highlights the importance of motivating children to work toward a goal through playful activities. Researchers have also studied the use of new technologies, such as virtual reality (VR), augmented reality, game consoles, and robots, which are familiar and appealing to children because they provide opportunities for play via interactive games while enriching the therapeutic experience [3].

Furthermore, these new technologies provide monitoring and automatic feedback on performance, the opportunity for repetition to improve motor skills, and sharing experiences between children and others. These are also financially accessible technologies that require low technical support, making them appropriate for use at home. Some examples of successful studies on hand therapy for children with CP and other motor disabilities are the studies conducted by Reid [6] and analyzed in the review by Pereira et al [7]. They concluded that VR is suitable for supporting hand therapy. Koutsiana et al [8] concluded in their review that serious games are an alternative to provide motivation in therapy. Moreover, Winkels et al [9] showed positive results in participants’ usability, user satisfaction, and enjoyment in gaming with the Nintendo Wii sports games, boxing, and tennis. In their review, Ayed et al [10] highlighted that the interest in the field of VR systems for rehabilitation is increasing. However, none of these studies provide an overview of the extent and range of the research on playful technological interventions. There does not seem to be a systematic approach to how and when we use technology and play in therapy for children. Many studies have experimented with available technologies that can be adjusted for therapy without paying much attention to the play elements that can be applied. Therefore, there is a need to have an overview of what the field has been doing for the last decade and to deepen our understanding of the use of play in therapy.

### Objectives

The range of playful technological interventions can be studied along multiple dimensions. First, the type of technology (motion sensing, game consoles, etc) can be used to categorize the research. Second, the type of playful elements (competition, rules, fantasy, etc) can help structure the analysis. Therefore, in this scoping review, we aim to identify which innovative technologies are part of playful hand therapies and what are the playful elements used in these interventions. This information will provide researchers, designers, and practitioners with an overview of current therapies for children with CP that make use of innovative technologies and play. Furthermore, we aim to provide starting points to design new therapies that are supported by innovative technology and play (which makes them suitable for other environments than just the rehabilitation center) and that engage and motivate children.

### Designing Playful Interactions

To analyze and determine whether and which playful elements have been applied in technology-supported hand therapies for children with CP, we used the Lenses of Play [11]. The Lenses of Play is a design toolkit used to create playful interactions. For example, Almahmoud [12] used the Lenses of Play to design a toy for children with autism. We have chosen to use it as our framework because it provides multidimensional examination of play beyond traditional therapist-centered approaches, making a distinction between games and free play [13] and providing a diverse set of playful elements such as control and competition among others. Other frameworks or models used in therapy, such as Theraplay [14] or SCOPE-IT [15], refer more to the behavior of children in connection with their caregivers and occupational performance. Although play is an essential element, these frameworks lack the focus on what makes an activity or object playful for children. The Lenses of Play focus on the object, game, and user interaction. This framework will help us to better understand how playfulness is used and identify the necessary ingredients to design new playful experiences for therapy. In the future design of playful therapies, this can lead to a common and more specific language to be used by the different stakeholders involved in innovative, technology-supported therapies, including clinicians, children, and their parents, as well as technology developers.

First, we briefly describe the technologies used in this review. Subsequently, identifying the playful elements used within therapies that use new technology will support a more systematic reflection and understanding of the potential benefits of using these technologies.

## Methods

A scoping review aims to compile the relevant literature and map the critical concepts of a specific topic. For this scoping review, we used the five-stage framework proposed by Arksey and O’Malley [16]: (1) identifying the research questions; (2) identifying relevant studies; (3) study selection; (4) charting the data; and (5) collating, summarizing, and reporting the results.

### Identifying the Research Questions

In line with the main objectives of this scoping review, we aim to answer the following research questions:

- Question 1. Which innovative assistive technologies are used in hand therapies for children with CP?
- Question 2. Which playful elements are embedded in the technology-supported therapy to motivate children with CP?

### Identifying Relevant Studies

To identify relevant studies, we performed comprehensive searches in the Scopus, Web of Science, and CINAHL databases for medical and human-computer interaction studies published in English between January 2009 and December 2022. The final search was performed on January 17, 2023. The keywords used were as follows: “cerebral palsy OR cerebral paresis OR cerebral palsies; AND play* OR game OR gamification OR toy; AND child OR children; AND therapy OR rehabilitation OR treatment; AND hand OR upper limbs.” The queries that were run on all the databases are presented in Multimedia Appendix 1.

### Study Selection

After removing duplicates, the titles and abstracts were analyzed according to the inclusion and exclusion criteria set by 2 reviewers (AB and TVPC). The inclusion criteria for the analyzed articles were as follows: (1) study participants were children (aged 0-18 years) with spastic CP; (2) the therapy described was focused on hands or upper limbs motor skills; (3) the therapy used innovative technology (ie, electronic tools, systems, and devices); (4) the therapy used playful elements such as video games or toys; and (5) the publications consisted of peer-reviewed academic articles or conference proceedings that were published between 2009 and 2022. The exclusion criteria included (1) insufficient information about the game or play activity or referred to traditional therapies without the support of technologies (eg, bimanual, constrained-induced movement, and Pirate Therapy), (2) lack of focus on the treatment used, (3) written in a different language than English, (4) unavailability to access the full text at the time of analysis, and (5) incomplete inclusion criteria. Disagreements between reviewers with regard to the exclusion criteria were resolved through discussion.

### Charting the Data

The data extracted from the selected studies included authors, date of publication, research questions or aim, sample size, frequency, time and duration of therapy, hand movement, intended environment (home or rehabilitation center), the technology used, description of the system, and playful elements according to the Lenses of Play.

### Collating, Summarizing, and Reporting the Results

The PRISMA-ScR (Preferred Reporting Items for Systematic Reviews and Meta-Analyses extension for Scoping Reviews) guidelines [17] were followed for reporting the results; the PRISMA-ScR checklist is included in Multimedia Appendix 2. The protocol was registered with the Open Science Framework. The authors (TVPC, BK, BS, and GL) met to determine the categories of technologies, evaluate the playful elements of the Lenses of Play, and analyze the extracted data. To determine the categories of technologies, we investigated their functionality and determined commonalities. For example, Kinect and Leap Motion are used to detect movement; hence, we created a motion sensor category. To identify the playful elements used in the interventions, we analyzed the information provided for each intervention through each of the Lenses of Play. To do so, we examined the design principles, play mechanisms, and goals that were included in the interventions and described in the paper. For commercially available games and technologies, we also investigated the information provided on the developers’ websites [18-23]. For example, when analyzing a study with the Lens of Play *Playful experiences*, if the intervention described a game where the player had to grab a specific number of virtual butterflies and place them in a jar, the game will be categorized under the playful experience *completion* because the player must complete a task. The same was performed with the other lenses by following the definitions of each element of the Lenses of Play.

## Results

### Overview

**Figure 1:**
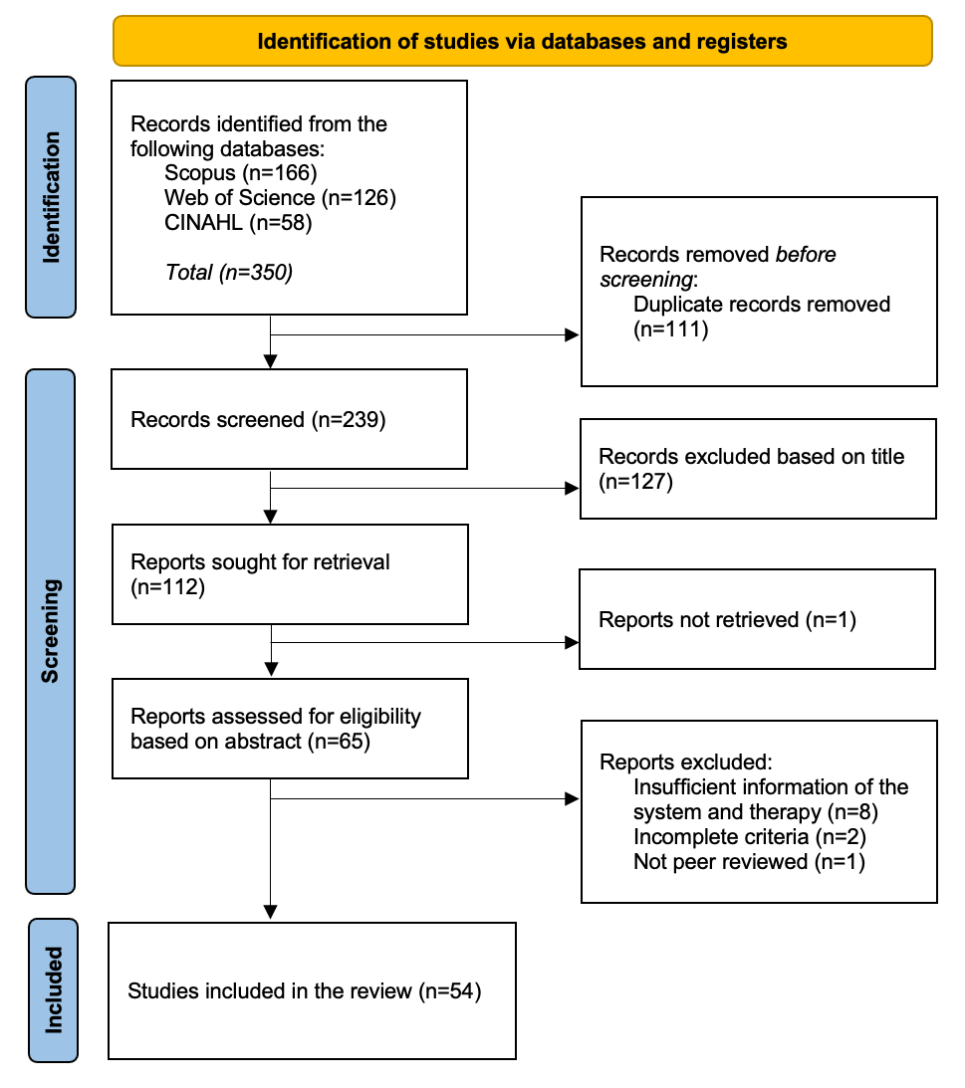
PRISMA (Preferred Reporting Items for Systematic Reviews and Meta-Analyses) flow diagram of search process.

The total number of studies found on Scopus was 166, whereas 126 studies were found on Web of Science (including MEDLINE) and 58 studies were found on CINAHL, resulting in 350 studies, including duplicates. The first author (TVPC) removed duplicate studies, resulting in 239 studies. The first 2 authors (TVPC and AB) conducted the analysis of titles and abstracts based on the inclusion and exclusion criteria. This yielded a total of 66 studies whose full text were reviewed for data extraction by 1 of the authors (TVPC). Figure 1 depicts the number of studies identified at each process stage following the PRISMA (Preferred Reporting Items for Systematic Reviews and Meta-Analyses) flow diagram. Finally, 54 studies were included in the study (Table 1).

**Figure 2:**
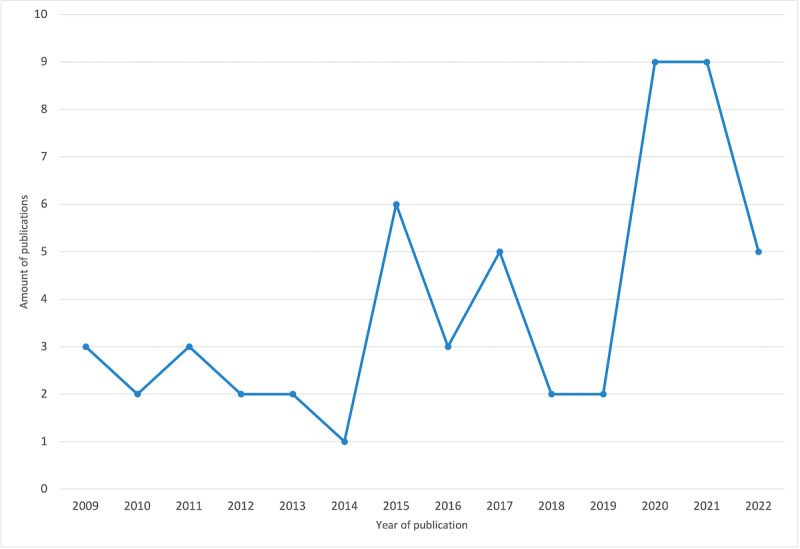
Publications per year.

In recent years, there has been an increase in the interest in developing and researching hand therapies that use innovative assistive technology and playful elements. Figure 2 shows the distribution of the selected papers from 1999 to 2022.

**Table 1:** Summary of analyzed studies and assistive technology (original purpose of the technology and type of hardware).

Study, year			Study				Technology
			Goal		Participants		Category and system
**Category 1: therapeutic-specific technology**							
	Bian et al [24], 2020	To develop toy modules in combination with Lego blocks to support hand and arm training		5 children, aged 5-10 years; 1 with amentia and 4 with hemiplegia		Category: Smart Tangibles (smart blocks) System: path building with the smart blocks and Lego blocks	
	Guberek et al [25], 2009	To evaluate the level of cooperation and satisfaction of children when practicing arm and hand movements during play-like activities in a physical environment		Children aged 5-12 years		Category: Motion Sensing (IREX^a^) System: the IREX system with the game Zebra Crossing, the child attempts to touch as many stars as possible while advancing the crosswalk	
	Mandil et al [26], 2015	To use a tangible user interface in designing tabletop activities to help motivate children with motor disabilities to increase the number of exercises and improve the motor proficiency and quality of life		4 children with CP^b^, aged 6-14 years; 3 physiotherapists		Category: Smart Tangibles (PhysiTable) System: PhysiTable with 3 different paths defined with LEDs. Music and color used for feedback. The player uses a cube to trace the path.	
	van Delden et al [27], 2012	To study the use of tangible, interactive games for the repetitive training of upper limbs in the therapy of children with CP		4 therapists; 16 children with CP, aged 2.5-8 years; 14 non-CP children aged 8-9 years		Category: Smart Tangibles (smart toys and TagTiles) System: the smart toys are used to manipulate the TagTiles	
	Wu et al [28], 2022	To develop an Interactive Story Box to facilitate rehabilitation of speech, cognition, and motion		4 children with CP, aged 4-8 years		Category: Smart Tangibles (interactive story box) System: Raspberry PI, RFID^c^ readers, and tangible objects to create an interactive box. Story maps are controlled with characters in the shape of a puzzle.	
**Category 2: consumer technology**							
	Acar et al [29], 2016	To investigate the efficiency of Nintendo Wii games in addition to neurodevelopmental treatment in patients with CP		30 children with CP, 16 female participants, aged 6-15 years		Category: game consoles System: Nintendo Wii, with the VR^d^ games (tennis, baseball, and boxing)	
	Avcil et al [30], 2021	To compare the effects of neurodevelopmental therapy and video game–based therapy for upper extremities		30 children with CP, aged 6-15 years		Category: game consoles (Nintendo Wii) and motion sensing (Leap Motion) System: 2 video games to improve hand and grip functions. “CatchAPet” to touch rabbits with repetitive wrist flexion or extension movements and “Leapball” to grasp a virtual ball with all fingers and throw it by finger extension.	
	Chen et al [31], 2021	To evaluate the feasibility of a Kinect-based constraint-induced therapy		10 children with CP in phase 1 and 8 children with CP in phase 2		Category: motion sensing (Kinect), computers System: video game where the child is a warrior defending their island. The Kinect detects the hand movements to throw cannonballs.	
	Chiu et al [32], 2014	To investigate whether Wii Sports Resort training is effective and if any benefits are maintained		62 children with hemiplegia aged 6-13 years		Category: game consoles (Nintendo Wii) System: Nintendo Wii, with Wii Sports Resort games, from easiest to hardest: bowling, Air Sports, Frisbee, and Basketball. 10 minutes per game.	
	de Oliveira et al [33], 2016	To develop a VR game using Unity 3D to aid motor and cognitive rehabilitation in children with CP		8 clinical experts		Category: computer (PC), motion sensing (Leap Motion), and wearables (Mind wave) System: 6 phases of a video game in a PC controlled by Leap Motion. Mind wave used to keep track of player’s attention.	
	El-Shamy and El-Banna [34], 2020	To investigate the effect of Wii training on hand function		40 children with hemiplegic CP, aged 8-12 years		Category: game consoles (Nintendo Wii) System: playing 4 Wii games: tennis, boxing, bowling, and basketball	
	Elsaeh et al [35], 2017	To develop a high-level control in which the human brain is stimulated by the visual, audio, and tactile sensation to transmit instructions to the affected upper limb’s joints		2 children with hemiplegia, 1 7 years old female participant, and 1 10 years old male participant		Category: computer (PC), controller (Novint Falcon) system: 3 video games on a PC using the Novint Falcon controller	
	Garcia-Hernandez et al [36], 2021	To examine how the subjective experience of seeing and controlling a half-body avatar or an abstract hand representation in a virtual environment for training upper limb movements may affect motor performance		19 children aged 7-9 years		Category: computer and motion sensing (Kinect) System: a video game with body or hand representation where participants have to reach and grab a ball	
	Gieser et al [37], 2015	To recognize and classify static gestures from Leap Motion by comparing classification techniques, decision trees, support vector machines, and k-nearest neighbors. Create and evaluate a game to detect hand gestures.		Volunteers and experts		Category: computer (PC) and motion sensor (Leap Motion) System: virtual game developed on Unity for PC and controlled via Leap Motion.	
	Golomb et al [38], 2009	To describe the learnings of providing home telerehabilitation to people with CP and to suggest ways to address some of the barriers to home telerehabilitation in this population		3 adolescents with CP		Category: console (PlayStation 3) and controller (5DTD Ultra sensing glove). System: custom games programmed in Java3D for PlayStation 3 and controlled with a 5DT5 Ultra sensing glove.	
	Golomb et al [39], 2010	To investigate whether in-home remotely monitored VR video game–based telerehabilitation in adolescents with hemiplegic CP can improve hand function and forearm bone health and demonstrate alterations in motor circuitry activation		3 patients with severe right hemiplegic CP, aged 13-15 years		Category: console (PlayStation 3) and controller (5DTD Ultra sensing glove). System: the 5DT5 Ultra sensing glove has 5 fiber optic sensors in each of the 5 fingers; it is connected to a PlayStation 3 with Linux, and the games were programmed using open source Java3D API^e^.	
	Goyal et al [40], 2022	To report the use of a VR gaming system and haptic feedback and its effectiveness		1 child, aged 6 years		Category: game consoles (PlayStation) System: a driving simulation game with PlayStation 4	
	Gregory et al [41], 2012	To enable play for children with CP that continuously entertains, which will allow extended play over long durations		N/A^f^		Category: smart tangibles (Pleo) and controller (Wii Nunchuk) system: a Wii Nunchuk is used to teach dance movements to Pleo.	
	Hernández et al [42], 2018	To test the usability of the gaming station with clinicians and children with CP and to establish the feasibility in a 12-week clinical trial		6 therapists and 6 children with CP, aged 7-16 years		Category: controller (Novint Falcon) System: force feedback Novint Falcon game controller, custom grips, arm and wrist supports, and software to be used with mainstream games	
	Hsieh et al [43], 2020	To improve hand performance while playing with Chinese puppets modified with Lego robots		42 children with CP		Category: smart tangibles (Lego Mindstorms NXT) System: modified puppets with Lego, using servo motors, sensors, and connecting cables	
	Hung et al [44], 2018	To study the feasibility and possible efficacy of a suite of motion-controlled games designed for upper-extremity training in children with CP using Kinect2Scratch		13 children with CP; mean age 6.9 years		Category: computer (PC), motion sensor (Kinect) system: 3 video games in a PC with a screen with a Kinect sensor Scratch visual programming and Kinect2Scratch software	
	Kassee et al [45], 2017	To compare a Nintendo Wii intervention to single-joint resistance training for the upper limb		6 children with spastic hemiplegic CP aged 7-12 years		Category: game Consoles (Nintendo Wii) System: experimental group: Nintendo Wii controllers, and selected games. Control group: TheraBand, Elite band and squeeze ball with a list of exercises.	
	Kottink et al [46], 2017	To assess the feasibility, in terms of gaming experience, a mixed-reality system for rehabilitation of the arm and hand function		5 children aged 7-12 years with CP and 10 adults aged 30-70 years with stroke or brain injury		Category: motion sensing (Kinect), computers (PC) System: HandsOn game—reaching, grasping, and releasing a physical object to control a PC video game using Kinect	
	Leal et al [47], 2020	To verify if there was any performance improvement in a task performed in a virtual environment and if it is transferable to the real environment		28 children with CP, aged 6-15 years		Category: motion sensing (Kinect), computers, and smart tangibles (touchscreen) System: Check Limit Game, pop bubbles with the touchscreen or gestures	
	Li et al [48], 2009	To assess if a low-cost VR therapy home-based system can promote movements of the hemiplegic upper extremity that the child finds difficult		5 children with CP aged 8 years		Category: motion sensing (EyeToy) and game console (PlayStation2) System: VR therapy home-based system that consists of video games (Secret Agent and Mr Chef) for PlayStation2 and controlled with EyeToy	
	Macintosh et al [49], 2020	To assess the feasibility of an intervention that combines a cocreated gaming technology with biofeedback and coaching		19 children, aged 8-18 years		Category; wearables (MYO Armband) and computers System: Dashy Square video game played with the use of electromyography and an MYO armband	
	Macintosh et al [50], 2022	To describe the design and evaluation of a biofeedback virtual game		9 children		Category; wearables (MYO armband) and computers System: Dashy Square video game played with the use of electromyography and an MYO armband	
	Nai et al [51], 2019	To analyze the use of Vive trackers to estimate forearm axial rotation for the purpose of supporting interaction with serious games		8 healthy participants aged 21-31 years		Category: motion sensing (HTC Vive trackers) and computers (PC) System: HTC Vive trackers attached to a wrist bracer to control a serious game system on a PC. One tracker used around the palm and another around the center of the forearm.	
	Pruna et al [52], 2017	Evaluate the use of a haptic device and VR games in upper limb rehabilitation in children		5 children with mild spasticity, aged 7-12 years; 4 children with Down syndrome and difficulty of movement in upper limbs, aged 9-12 years		Category: controller (Geomagic Touch) and VR headsets (Oculus Rift) System: 2 interactive virtual environment games (watering plants and order objects). The haptic device (Geomagic Touch) acquires the movement generated by the user, and an Oculus Rift provides immersion in the use of the system.	
	Stansfield et al [53], 2015	To further investigate whether improved measures of motor performance will be seen with the use of motion-based VR gameplay		1 boy aged 10 years		Category: computers (PC) and wearables (Polhemus Liberty) System: PC with the Polhemus Liberty tracking sensor, a screen, and a memory game played alone, in cooperation or competition	
	Tarakci et al [54], 2020	To study the potential efficacy of an 8-week program with the Leap Motion controller-based training as a therapeutic method for upper-extremity rehabilitation in comparison with conventional rehabilitation programs in children with CP, juvenile idiopathic arthritis and brachial plexus birth injury.		Group 1 (CP: n=15; JIA^g^: n=18; and BPBI^h^: n=9). Group II (CP: n=15; JIA: n=25; and BPBI: n=10). Aged 5-17 years.		Category: motion sensing (Leap Motion) and computers (PC) System: 2 rehabilitative video games on PC using Leap Motion: Fizyosoft CatchAPet and Fizyosoft Leapball	
	Winkels et al [9], 2013	To explore the effect of the Nintendo Wii training on upper-extremity function in children with CP		15 children with CP		Category: game consoles (Nintendo Wii). System: children played the boxing and tennis games provided in the Nintendo Wii Sports video game console.	
	Yildirim et al [55], 2021	To investigate the effect of leap motion–based exergame therapy		20 children with CP, aged 8-15 years		Category: motion sensing (Leap Motion) and computers System: 2 video games controlled with a leap motion. Leap Ball: grab a ball and throw it into a box of matching color. Catch a Pet: touch the moles in a certain order	
	Zoccolillo et al [56], 2015	To investigate the effectiveness of video game therapy with respect to conventional therapy in improving upper limb motor outcomes		22 children with CP, aged 4-14 years. GMFC^i^ between I and IV.		Category: game consoles (Xbox) and motion sensing (Kinect) System: a videogame of the console Xbox using the Kinect device for motion capture. Six games available: “Space pops,” “20.000 Leaks,” “Rally Ball,” boxing, volley, and bowling.	
**Category 3: therapeutic and consumer technology**							
	Amonkar et al [57], 2022	To evaluate the feasibility of implementation, acceptance, and perceived efficacy of a joystick-operated ride-on-toy intervention to promote upper-extremity function		11 children with CP, aged 3-14 years; 11 caregivers; and 6 clinicians		Category: joystick ride-on-toy System: children rode the ride-on-toy (car) navigating with the spastic hand and performing a task throughout the path (collecting objects, throwing balls, and avoiding obstacles)	
	Bortone et al [58], 2020	To determine the efficacy of immersive virtual environments and wearable haptic devices		8 children with CP or developmental dyspraxia		Category: wearable (haptic device for the fingertip) and VR headsets (Oculus Rift VK2) System: collecting coins in a VR environment and placing them in a moving piggy bank. Slide a token out of a virtual labyrinth with the finger. Difficulty changes with time.	
	Choi et al [59], 2021	To investigate the efficacy of a VR rehabilitation system of wearable multi-inertial sensors for upper limb		80 children, aged 3-16 years with brain injury including CP		Category: wearable (Neofect Smart Kids) and computers System: games with activities of daily living promoting wrist and forearm articular movements using wearable inertial sensors.	
	Cifuentes-Zapien et al [60], 2011	To study if a video game can be used as an interface for a robot for the rehabilitation of the pronation and supination movements of children with CP		1 healthy right-handed child aged 11 years.		Category: computers (PC) and robotics (robotic arm). System: a PC video game developed for an upper limb rehabilitation robot for children with CP. The video game simulates a formula one race car on a racetrack. The car’s horizontal position is controlled by the pronation and supination motions.	
	Crisco et al [61], 2015	Evaluate play activity recorded by the controller for 2 toys and 3 computer games.		20 children aged 5-11 years		Category: controller (arm and elbow remote), smart tangibles (smart toy: car and dog), and computers (PC). System: a specially designed arm and elbow remote controller was used to interface wirelessly with 2 smart toys. System: A specially designed arm and elbow remote controller was used to interface wirelessly with 3 video games.	
	Crisco et al [62], 2015	To develop and evaluate the measurement accuracy of innovative, motion-specific play controllers that are engaging rehabilitative devices for enhancing therapy and promoting neural plasticity and functional recovery in children with CP		6 typically developed children (3 male participants and 3 female participants aged 5-11 years)		Category: controller (arm and elbow remote) and smart tangibles (smart toy: car) System: the play arm and elbow remote controller was designed with a conformable, ergonomic handle to accommodate varying levels of contractures among children with CP and control a car.	
	Dunne et al [63], 2010	To describe the hardware platform, present the design objectives derived from iterative design phases and meetings with clinical personnel, and discuss the current game designs and identify areas of future work		Expert clinicians on CP		Category: wearables (accelerometer) and smart tangibles (multitouch display, tangible objects). System: 3 games played (Find the bone, Spelling, and Catch the butterflies); the tangible objects control the game in a multitouch display. An accelerometer measures body changes and modifies the game, for example, butterflies fly off the jar.	
	Fu et al [64], 2020	To determine if children could tolerate 9 laboratory treatment sessions and administer up to 7.5 h/wk of CCFES^j^ video game therapy at home		3 children aged 8-11 years with hand hemiplegia		Category: computer, wearables (arm sensors and electrical stimulation electrodes) System: 4 video games (Paddle Ball, Sound Tracker, Skee-Ball, and Marble Maze)	
	Hernandez et al [65], 2021	To explore the effectiveness of interactive computer play with haptic feedback		13 children with CP, aged 7-16 years		Category: controllers (Novint Falcon and Custom levers) and computers System: 4 commercial video games to train wrist movement: Crazy Rider, Swooop, Funky Karts, and Lil Mads and the Gold Skull. 5 video games to train elbow and shoulder movement: Looney Tunes Dash, Heroes of Loot, Bird Brawl, Pac-Man, and Save the Day.	
	Minh et al [66], 2021	To test a design of 2 interactive toys and an open game		4 children with CP, aged 2-3 years		Category: computers and smart tangibles (smart toys) System: a stuffed stick toy with a 6-DOF inertial measurement unit (IMU) and force sensor–incorporated gloves to squeeze a ball used to play “Catch the worms in the garden”	
	Mirich et al [67], 2021	To assess the efficacy of VR rehabilitation		1 child aged 4 years		Category: wearables (Neofect Smart Kids) and computers System: a functional activity game with different activities such as turn pages, painting, wiping a table, and playing ping pong selected based on the needs of the patient	
	Mittag et al [68], 2020	To present the design and implementation of a tangible device for hand training		N/A		Category: wearables (arm sensors) and computers System: a video game controlled by the sensors and interactions with the tangible controller.	
	Parmar et al [69], 2021	To improve rehabilitation programs for children and adults with neurodevelopmental disorders in a game-based telerehabilitation.		6 children with CP; 10 adults recovering from a stroke		Category: wearables (motion therapy mouse) and computers System: the motion mouse is attached to different objects such as a ball, to control movement in a commercial video game (Big Fish Game).	
	Peper et al [70], 2013	To examine the potential effects of the training on bimanual coordination and identify if the training had beneficial effects on the affected arm’s performance		6 children with CP aged 7-12 years		Category: controller (custom levers) and computers (laptop) System: 2 horizontal levers, a laptop computer, and an additional monitor. Left-hand movements produce vertical displacements, and right-hand movements produce horizontal displacements.	
	Preston et al [71], 2016	To study the feasibility of using computer-assisted arm rehabilitation computer games in schools, their preference for single player or dual player mode, and changes in arm activity and kinematics		9 boys and 2 girls with CP aged 6-12 years; mean age 9 years		Category: robotics (robotic arm) and computers (PC) System: an assistive robotic arm connected to a computer with cooperative and competitive games	
	Psychouli et al [72], 2017	To propose a system that can enhance children’s motivation during the implementation of a CIMT^k^ session and that could explore differences in compliance rates, motivation levels, and intervention feasibility		Non-CP children and 3 groups of CP children (CIMT, RT^l^, and CIMT+RT), aged 5-11 years		Category: wearables (arm sensors) and smart tangibles (smart toys) System: arm and hand with sensors (accelerometer, magnetometer, gyroscope for upper and lower arm, and flex sensors on the wrist and fingers) that control the 4-wheeled robotic vehicle (DFRobot Cherokee)	
	Sabry et al [73], 2020	To develop a low-cost VR rehabilitation system with a data glove and virtual games		8 children with CP, aged 5-12 years		Category: wearables (data glove) and computers System: a data glove is used to play video games: “Grasp the ball”	
	Stroppini et al [74], 2022	To determine the feasibility and efficacy of the MusicGlove to motivate hand function		3 children with hemiparetic CP, aged 6-17 years		Category: wearables (MusicGlove) and computers System: a video game is controlled with the glove. The patients tap their fingers to make musical notes according to the notes that show up on the screen.	
	van Loon et al [75], 2011	To test a set of video games, developed to loosen the coupling between the hands of children with CP		7 children with spastic unilateral CP, aged 7-12 years		Category: controller (custom levers) and computer (PC) system: 3 computer games that challenged the participants to move their hands according to 6 different bimanual coordination patterns executed with custom levers.	
	Weightman et al [76], 2011	To compare upper limb kinematics of children with CP using a passive rehabilitation joystick with adults and able-bodied children to better understand the design requirements of computer-based rehabilitation devices		9 adults (aged 23-30 years), 9 children (aged 7-9 years), and 7 children with CP (aged 5.5-7 years)		Category: a controller (joystick) and computers (PC) System: a computer game in which the child controlled a “spaceship” collecting “satellites” with the use of a joystick.	

^a^IREX: immersive rehabilitation exercise.

^b^CP: cerebral palsy.

^c^RFID: radio frequency identification.

^d^VR: virtual reality.

^e^API: application programming interface.

^f^N/A: not applicable.

^g^JIA: juvenile idiopathic arthritis.

^h^BPBI: brachial plexus birth injury.

^i^GMFC: gross motor function classification.

^j^CCFES: contralaterally controlled functional electrical stimulation.

^k^CIMT: constraint-induced movement therapy.

^l^RT: robot-assisted therapy.

### Innovative Assistive Technology

The authors identified 2 ways to classify the innovative assistive technologies used in the analyzed studies: the original purpose of the technology and the type of hardware.

#### Original Purpose of the Technology

This first type of classification refers to therapeutic-specific technology, consumer technology, and the combination of therapeutic and consumer technology. Table 1 shows the details of the studies per category. Therapeutic-specific technology was developed for the sole purpose of being used in hand therapy and has been used in 5 studies. An example of this type of technology is the immersive rehabilitation exercise, which is a video gesture control technology that allows patients to be immersed in a video game where users can exercise by interacting with the elements of the game [25]. Consumer technology is a commercially available technology used in entertainment or other fields but that has been modified to be used in hand therapy, consumer technology was used in 29 studies. The authors of the included studies identified this type of technology as potentially beneficial and motivating because of its existing functionalities, interactions, familiarity, and lower costs [54]. For example, Acar et al [29] investigated the efficiency of Nintendo Wii games together with neurodevelopmental treatment in patients with CP. They analyzed “out-of-the-box” games, such as tennis, baseball, and boxing, focusing on the upper extremities. In addition to the observed improvements in speed and functional independence, the children perceived the use of the Nintendo Wii as a reward. The rest of the studies (20 in total) made use of a combination of therapeutic-specific technology and consumer technology; an example is presented in the studies by Golomb et al [38,39], where custom games were developed to be used with the PlayStation 3 console (consumer technology) in combination with the 5DT Ultra sensing glove (therapeutic-specific technology). The games encouraged hand movements such as opening and closing or thumb extension; speed was also trained by challenging the player to chase a butterfly by flexing or extending the fingers rapidly.

#### Types of Hardware

This second type of classification refers to a more general category of hardware or technologies, such as VR headsets, game consoles, wearables, motion sensing, controllers, smart tangibles, and robotics (Table 2). When analyzing the types of hardware, it was found that the most common technologies used were computers (32/54, 59%), as they were often used to deploy a video game and to connect with other types of technology. Wearables were one of the most used technologies (19/54, 35%); wearables are continuously in close contact with the body to capture the movement of the hand or arm while they can provide direct haptic feedback. Some of these technologies include gloves such as 5DT Ultra sensing gloves, the Neofect Smart Kids, the Data glove, and the Music glove. Smart tangibles were used in 15 studies, including smart toys such as the TagTile, a device similar to an electronic board game [27], and the dancing dinosaur Pleo! Dance! [41]. Motion-sensing technology was also frequently used (14/54, 25%); a device that belongs to this category is the Kinect, which was used in the studies by Hung et al [44], Kottink et al [46], and Zoccolillo et al [56] because of its ability to capture the upper extremities and movements of the users from a distance. Another widely used type of hardware was controllers (11/54, 11%), for example, the Novint Falcon, a haptic device that acts as a controller similar to a computer mouse but with a shape that allows for higher degrees of freedom. This device, which allows for resistive force feedback on the spastic hand, was used by Elsaeh et al [35] and Hernández et al, [42,65], where limitations of movements and direction are adapted to the interactions needed in the virtual games used and the possibilities of the spastic hand. The full list of types of hardware used in the studies can be found in the Multimedia Appendix 3 [9,24-76]. Some studies focused on using only one type of innovative assistive technology. In contrast, most studies (43/54, 80%) used a combination of ≥1 type, such as PCs with robotic arms [60], smart toys with an arm and elbow remote controller and a PC [61], wearables and smart tangibles [63], and custom levers with a PC [75]. The complete list of studies that used a combination of different types of hardware is provided in Multimedia Appendix 4 [27,30,31,33,35-39,41,42,44,46-76].

**Table 2:** Technology classification based on type of hardware.

Type of hardware and name		Value, n (%)
**Computers (n=32)**		
	PC	26 (81)
	Laptop	4 (17)
	Tablet	2 (6)
**Controllers (n=11)**		
	Custom levers	3 (27)
	Geomagic Touch	1 (9)
	Joystick	2 (18)
	Motion Therapy mouse	1 (9)
	Novint Falcon	3 (27)
	Wii Nunchuk	1 (9)
**Game consoles (n=11)**		
	Nintendo Wii	6 (55)
	PlayStation	4 (36)
	Xbox	1 (9)
**Motion sensing (n=14)**		
	EyeToy	1 (7)
	HTC Vive trackers	1 (7)
	IREX^a^	1 (7)
	Kinect	6 (43)
	Leap Motion	5 (36)
**Robotics (n=2)**		
	Robotic arm	2 (100)
**Smart tangibles (n=15)**		
	Interactive Story Box	1 (7)
	Lego Mindstorms NXT	1 (7)
	Multitouch display	2 (13)
	Pleo!	1 (7)
	PhysiTable	1 (7)
	Ride-on-toy	1 (7)
	Smart blocks	1 (7)
	Smart toys	5 (33)
	TagTiles	1 (7)
	Tangible objects	1 (7)
**VR^b^ headsets (n=2)**		
	Oculus Rift	2 (100)
**Wearables (n=19)**		
	5DT sensing gloves	2 (10)
	Accelerometer	1 (5)
	Arm and elbow remote	2 (10)
	Arm sensors	4 (21)
	Electrical stimulation electrodes	1 (5)
	Data glove	1 (5)
	Mindwave	2 (10)
	Music glove	1 (5)
	MYO Armband	2 (10)
	Neofect Smart Kids	2 (10)
	Polhemus Liberty	1 (5)

^a^IREX: immersive rehabilitation exercise.

^b^VR: virtual reality.

### Lenses of Play

#### Overview

**Figure 3:**
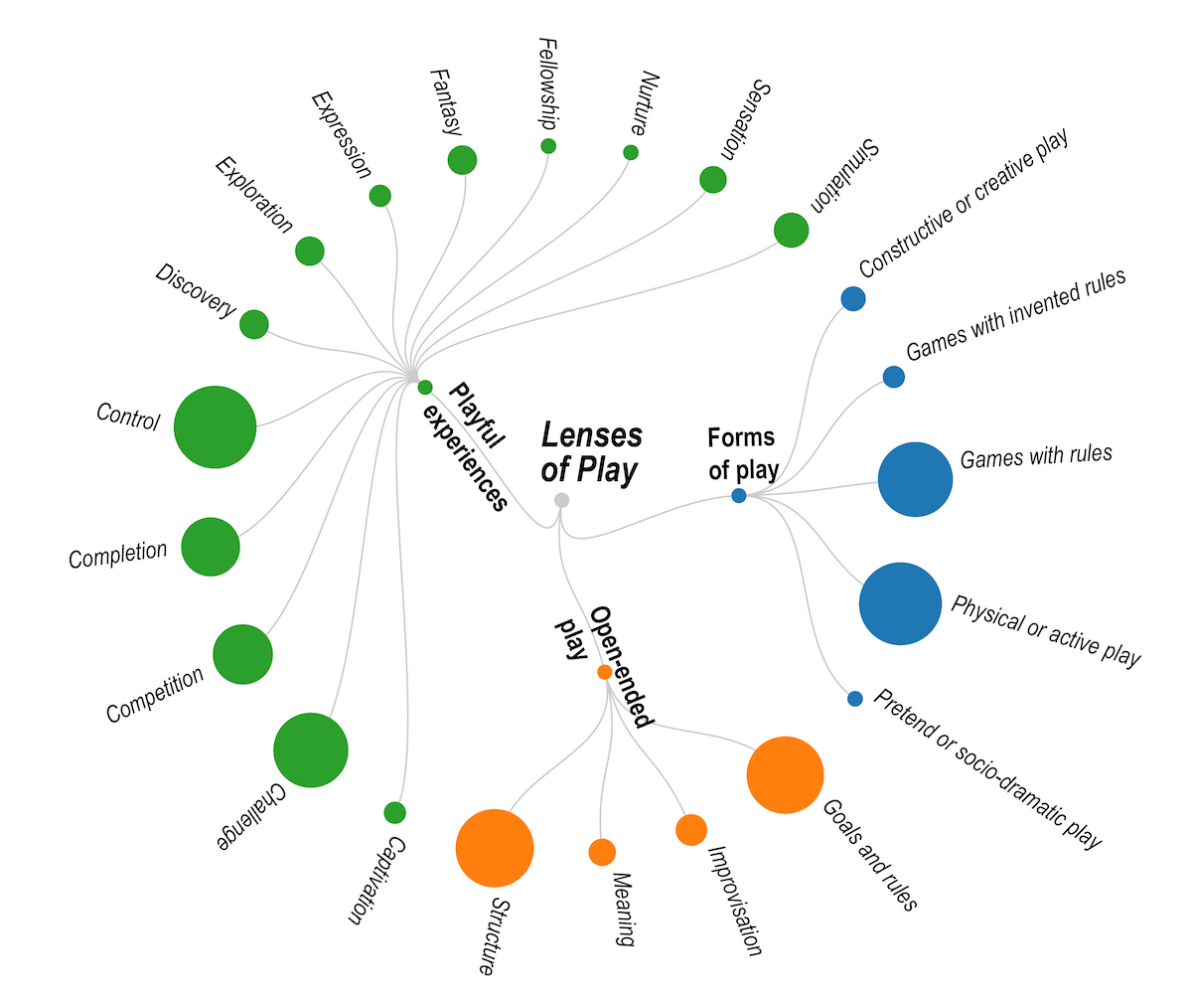
Lenses of Play; the size of the circles represents the frequency of use of the play element in the publications.

Bekker et al [11] defined the Lenses of Play as a toolkit that includes different perspectives on play that can inform design decisions throughout the design process. Initially, 4 lenses were defined: *Open-ended Play*, *Forms of Play*, *Stages of playful interactions*, and *Playful experiences*. In a later publication, Bekker et al [77] introduced a fifth lens, *Emergence*, which relates to the system’s perspective and how it can provide meaningful interactions. This analysis focuses on the *Open-ended Play*, *Forms of Play*, and *Playful experiences* (Figure 3) lenses, and the *Stages of playful interactions* and *Emergence* lenses have been omitted because little information was provided about these aspects of play in the studies included in this review. It was possible to identify the playful elements used in the proposed interventions for the other lenses (Table 3).

**Table 3:** Summary of analyzed studies by Lenses of Play.

Lens and play element			Categories				
			Therapeutic-specific technology		Consumer technology		Therapeutic and consumer technology
**Open-ended Play**							
	Improvisation	[24]		[41,43]		[57,61,62,67,72]	
	Structure	[25-28]		[9,29-40,42,44-56]		[57-61,63-71,73-76]	
	Goals and rules	[25-28]		[9,29-40,42,44-56]		[57,58,60-65,67-71,73-76]	
	Meaning	[24]		[41,43,52]		[61,72]	
**Forms of Play**							
	Constructive or creative play	[24,28]		[41,43]		[74]	
	Pretend or sociodramatic play	—^a^		[41,43]		—	
	Physical or active play	[24-28]		[9,29-56]		[57-76]	
	Games with rules	[25-27]		[9,29-40,42,44-52,54-56]		[57-61,63-65,68-71,73-76]	
	Games with invented rules	—		[28]		[61,62,72]	
**Playful experiences**							
	Captivation	[28]		[41,43,52]			
	Challenge	[25-27]		[9,29-39,42,44-56]		[57-61,63,64,67-76]	
	Competition	[24-27]		[9,29-34,40,42,44,45,47,48,51,53,55,56]		[58,61,63-65,67-71,74]	
	Completion	[26-28]		[30,31,35-37,40,47,49-53,55]		[57-59,64-67,69,71,74-76]	
	Control	[24-28]		[9,29-56]		[57-76]	
	Discovery	[24,28]		[33,43]		[61,62,72]	
	Exploration	[24,26]		[33,41,43,46]		[61]	
	Expression	[24]		[41,43]		[72]	
	Fantasy	[28]		[41-44]		[70,76]	
	Fellowship	—		[53]		[71]	
	Nurture	—		[41]		[70]	
	Sensation	—		[35,42]		[58,59,72,74]	
	Simulation	—		[35,40,42,44,45,48,56]		[60,63,74]	

^a^No articles in this category make use of this play element.

#### Lens 1: Open-Ended Play

According to Bekker et al [11], “Open-ended play can be positioned somewhere between paidia and ludus.” In paidia or free play, there is no end goal; it is chaotic, and there is space for improvisation and spontaneity, allowing for expression, meaning, and playing for its sensation. At the same time, ludus refers to games with fixed rules, structure, end goals, and challenge or competition. The *Open-ended Play* lens refers to the 4 properties that characterize paidia and ludus: improvisation, structure, goals and rules, and meaning, as defined by de Valk et al [78]. Players can create their own game during *improvisation* without preparation, whereas *structure* leaves little room for chaos or spontaneity in a game. If *goals and rules* have been defined, the game will have a finite status with a fixed process. Finally, players can add *meaning* to the interaction possibilities that the designers have established.

As shown in Table 3, most interventions presented in the articles have characteristics that refer to ludus, a structured form of play (49/54, 90%) with defined goals and rules (48/54, 88%). In contrast, elements that refer to paidia, such as improvisation (8/54, 15%) and meaning (6/54, 11%), were found in fewer studies. For example, Dunne et al [63] used 3 video games—Find the bone, Spelling, and Catch the butterflies—on a multitouch screen where tangible objects are used to play the games. An accelerometer measures body changes that indicate changes in inclination and use of compensatory movements. Changes in body posture modify the game, for example, butterflies fly off the jar. The 3 games have a clear structure and set of rules (ludus) that the player must comply with to reach a goal, such as catching all the butterflies inside a jar.

In contrast, Crisco et al [61] proposed a system where a robotic car and a robotic dog were controlled using a play controller. Children can play with the toys by performing different wrist motions in this system. There is no set of rules (paidia) besides the type of movements the controller allows. The interaction with the toys is not fixed and can rapidly change according to the children’s interests (improvisation and meaning).

#### Lens 2: Forms of Play

This lens refers to children’s development of skills through different forms of play. There are 4 primary forms of play according to Bekker et al [11]; a description of the forms is provided in Table 4. All the analyzed papers refer to an intervention with a physical or active form of play as they all aim to provide physical therapy for the players. Most interventions also presented games with rules (45/54, 83%); the only interventions that did not have games with rules were those that had more of an open-play approach assisted with a toy for example [24,28,41,61,62]. The intervention proposed by Gregory et al [41] contains 3 forms of play, constructive or creative play, pretend or sociodramatic play, and physical active play, where the player interacts with Pleo! Dance! the dinosaur and teaches it to dance using a remote control.

**Table 4:** Properties of Play Lens 2: Forms of Play.

Forms of play	Description [11]
Constructive or creative play	Creating and constructing something from objects.
Pretend or sociodramatic play	Acting out roles, often using toys and props.
Physical or active play	Sensory motor play with objects. In preschool years, this may involve rough-and-tumble play. Older children engage in play with a more vigorous component to test strengths and skills.
Games with rules	Playing games in social groups with fixed predetermined rules
Games with invented rules	Playing games with modified or rule sets invented

#### Lens 4: Playful Experiences

According to the Play Lens 4: *Playful experiences*, there are 20 different types of playful experiences: captivation, challenge, competition, completion, control, discovery, eroticism, exploration, expression, fantasy, fellowship, nurture, relaxation, sadism, sensation, simulation, subversion, suffering, sympathy, and thrill. We identified 13 out of the 20 playful experiences in the included studies, as described in Table 5. Control was the most used playful experience (the complete summary can be seen in Table 3). Control could be found in interventions that relied on structure and rules and in those with a more open-play form. For example, the study by Gregory et al [41] consisted of playing and teaching a dinosaur to dance, where playful experiences of captivation, control, exploration, expression, fantasy, and nurture could be found. At the same time, other interventions relied on more competitive (29/54, 54%) and challenging (45/54, 83%) experiences against avatars in the system, another player, or oneself, similar to those in Wii Sports [9,29,30,32,34,45].

**Table 5:** Properties of Play Lens 4: Playful experiences.

Type of Playful experiences	Description
Captivation	Experience of forgetting one’s surroundings
Challenge	Experience of having to develop and exercise skills in a challenging situation
Competition	Experience of victory-oriented competition against oneself, opponent, or system
Completion	Experience of completion, finishing and closure, in relation to an earlier task or tension
Control	Experience power, mastery, control, or virtuosity
Discovery	Experience of discovering a new solution, place, or property
Exploration	Experience of exploring or investigating a world, affordance, puzzle, or situation
Expression	Experience of creating something or expressing oneself in a creative fashion
Fantasy	Experience of make-believe involving fantastical narratives, worlds, or characters
Fellowship	Experience of friendship, fellowship, communality, or intimacy
Nurture	Experience of nurturing, grooming or caretaking
Sensation	Meaningful sensory experience
Simulation	Experience of perceiving a representation of everyday life

## Discussion

### Principal Findings

This scoping review aimed to provide an overview of the innovative technologies used in hand therapies for children with CP and to identify the playful elements used in such interventions. The 54 analyzed studies showed that therapists and researchers are investigating a broad diversity of technology combined with play to make therapy more pleasant, engaging, and effective for children and to overcome some of the challenges and needs encountered in hand therapy such as the lack of personalization; monitoring of progress; high cost of devices; and the need for tailored feedback, increased adherence, and motivation.

The results of this study show high use of consumer technologies such as the Nintendo Wii, PlayStation, Leap Motion, and several smart toys, to name a few, as a response to the need for financially accessible technology for therapy. This can help lower the therapy costs while allowing to practice in different contexts, such as at home or at school. In addition, considering the familiarity and interest children already have with these types of technology, they could be more easily accepted and adopted. Another important finding was the variety of technologies used; we identified at least 41 different devices. These devices come with a diversity of characteristics such as motion tracking (Kinect, Leap Motion, immersive rehabilitation exercise, etc) and haptic feedback (smart tangibles, TagTiles, Geomagic Touch, controllers, etc) or can be more general support systems (PC, laptop, and game consoles) that can easily be used to deploy a video game. Interestingly, 80% (43/54) of the studies used a combination of hardware; for example, de Oliveira et al [33] used Leap Motion in combination with Mindwave and custom software developed for the explicit purpose of therapy. The combination of commercial and custom hardware brings together the strengths of a robust technology that requires low technical support and can be used at home without extensive financial burden, with the requirements that specific treatments can bring.

From our analysis of play elements, we see different approaches to how play has been implemented and the type of technology used to enhance motivation and adherence: via video games, where the participants had to execute an action with their hands to advance in the game, and via toys or other types of tangible devices that allowed for a more open form of interaction to engage the user while performing the exercises. The findings show that under Play Lens 1: *Open-end Play*, “structure” and “games and rules” were most used in video games. The opposite can be observed in games with toy-like tangibles such as Pleo [41], TagTiles [27], Lego Mindstorm [43], and PhysiTable [26], which allow for a more open-play approach, where there is a structure of the game in terms of narrative. Yet, there is plenty of space for the players to explore and create their own rules, adding space for improvisation and meaning. Although not all the studies reported on motivation and improvement of skills, those that did showed that play has a vital role in motivating children to do their therapy while helping them improve their motor skills. This further supports the idea that *play* is a valuable factor in therapy because it appeals to children. Once they are in a state of flow or immersion in the game, they can perform different hand movements with repetition without becoming burdensome. Moreover, they can share their experience and create bonds through play with other children and family members.

One anticipated finding was that all the studies fit into the Play Lens 2: *Form of Play*, “physical or active play,” which refers to sensory motor play with objects and the test of strength and skills, which is paramount in physical therapy. Furthermore, there should be a flexibility in this “physical or active play” to be personalized to the skill level of children with CP and the use of technology can provide this personalization through the use of sensors and intelligent systems. The other *Form of Play* that was identified the most was “games with rules,” where participants often encounter a predefined goal and a clear set of rules that they had to comply with to play the game. Control (54/54, 100%) and challenge (45/54, 83%) were the most commonly used Play Lens 4: *Playful experiences*. They are often characteristics of video games and are also found in traditional therapy with toys guided by a therapist. The commonality of these approaches is that the participant had to accomplish a task by controlling an object in the virtual environment or a physical object. This control was strongly linked with the exercise that had to be performed with the spastic hand. In the case of therapy at home, it is challenging to achieve the guidance that therapists provide, but the use of technology can help solve this problem [31].

### Design New Therapies Supported by Innovative Technology and Play

From this analysis, we cannot draw strict conclusions regarding the impact of using one technology over another. We are also left with questions on how best to use open play or games with goals (Play Lens 1) and how to choose a specific play experience (Play Lens 4). Nonetheless, the playful elements and technology inventory gathered here can be a starting point for designers, researchers, and clinicians who wish to develop new interventions for children with CP. Researchers and designers should first be aware of the consequences and affordances of using one specific technology and the type of play they want to provide with the intervention. The analyzed interventions present toys and video games that offer different types of play and play experiences with their advantages and disadvantages. Although commercially available video games can be easier to develop and access, they might not fit the child’s specific level of disability.

In some cases, combining such technology with specialized software can provide better training. In contrast, toys can provide haptic feedback, allow object manipulation with both hands, and provide more freedom in the type of play, giving room for creativity. When designing new interventions, it is essential to consider certain aspects such as the type of play experience, level of challenge, competition, and physical activity. This kind of play experience could be games with rules or open play. The level of challenge should not frustrate the child but keep them motivated. With competition, we can provide the possibility to compete against themselves or someone else.

Further research can include comparing the effectiveness of different play strategies (for motivation and improvement of motor skills) and designing new interventions that use smart toys or other physical and digital play tangibles to broaden the knowledge base. Another interesting area of focus would be to study in more depth how hand therapy for children with CP can benefit from using new technologies, including artificial intelligence. The possibilities of using artificial intelligence in this area are broad; four examples are as follows: (1) the application of artificial intelligence for personalization (tailored to the skills of the user), (2) adaptive play complexity (changing complexity depending on the progress made in therapy or skills), (3) balanced play (the skills of different participants are leveled out), and (4) the use of data to help the therapists and caregivers to provide adequate support to the patients or create competition within a community to develop a shared experience. Many opportunities can make the experience of hand therapy more entertaining for children, and the combination of technology and play is a direction that can help achieve this.

### Strengths and Limitations

This paper provides a comprehensive review of the current state of hand therapy for children with CP, with a focus on the use of innovative technologies and playful elements. With this review, we have synthesized a wide range of the literature and identified key technologies and approaches in the field. We are also proposing a novel approach by including the Lenses of Play to analyze and understand the application of playful elements in technology-supported hand therapy. This framework provides a new perspective on play and offers a diverse set of elements that can inform the design of new therapies.

We acknowledge that this scoping review has limitations, and first is the lack of details of the interventions. The Lenses of Play allowed us to examine the papers from different perspectives. However, because the main goal of the research papers was to study the effectiveness of the interventions or propose new types of interventions, they did not contain a complete description of all the features of the games or toys, their design process, and the play experience. This could have added bias because we relied on the limited information provided in the studies and on the experience and knowledge of our team and the publicly available information of some of the technologies. Another limitation is the lack of scientific publications on other games, toys, and commercially available technology that therapists include in their program. A further limitation is that we only included studies written in English, excluding studies published in other languages.

### Conclusions

With this scoping review, we found that the role of play in the interventions that use innovative technologies in hand therapy for children with CP is to create an enjoyable activity for children that can also help them improve their motor skills. We have provided an overview of how and which technologies are available and which playful elements are part of the interventions. Currently, the field shows diverging strategies and a variety of playful experiences that are supported by technology. Whether via a video game or a toy, with rules and structure, or open play, repetition becomes fun and engaging. While playing, children enjoy the activity and forget that they are performing repetitive hand movements. Technology is an appealing medium to support play; it provides advantages such as measuring hand and arm movement and integrating them in the interaction with the games. In addition, it has functionalities for personalization according to the degree of spasticity of the child and their personal preferences. Technology can also provide feedback to guide the therapy and the game and improve the play experience while collecting valuable data for the therapists, the caregivers, and the children. The implementation of personalized and adaptive therapies in the home or school environment can help relieve the workload on the caregivers and rehabilitation centers. Together with play, innovative, assistive technologies provide an intrinsic incentive to exercise and continue with therapy.

## Appendix

Multimedia Appendix 1 Search queries.

Multimedia Appendix 2 PRISMA-ScR (Preferred Reporting Items for Systematic Reviews and Meta-Analyses extension for Scoping Reviews) checklist.

Multimedia Appendix 3 Types of hardware and studies.

Multimedia Appendix 4 Studies that use a combination of different types of hardware.

## Abbreviations

CP: cerebral palsy
PRISMA: Preferred Reporting Items for Systematic Reviews and Meta-Analyses
PRISMA-ScR: Preferred Reporting Items for Systematic Reviews and Meta-Analyses extension for Scoping Reviews
VR: virtual reality
