# Supplementary material for: Playfulness and New Technologies in Hand Therapy for Children With Cerebral Palsy: Scoping Review
Source: JMIR Serious Games. 2023 Oct 16;11:e44904. doi: 10.2196/44904 (PMC10616756; doi:10.2196/44904)
Supplement: Multimedia Appendix 1 [file games_v11i1e44904_app1.docx]

## Multimedia Appendix 1 – Search query

### Scopus

( TITLE-ABS-KEY ( "cerebral palsy" OR "cerebral paresis" OR "cerebral palsies" ) AND TITLE-ABS-KEY ( play* OR game OR gamification OR toy ) AND TITLE-ABS-KEY ( child* ) AND TITLE-ABS-KEY ( therapy OR rehabilitation OR treatment ) AND TITLE-ABS-KEY ( hand OR "upper limbs" ) ) AND ( LIMIT-TO ( PUBYEAR,2022) OR LIMIT-TO ( PUBYEAR,2021) OR LIMIT-TO ( PUBYEAR,2020) OR LIMIT-TO ( PUBYEAR,2019) OR LIMIT-TO ( PUBYEAR,2018) OR LIMIT-TO ( PUBYEAR,2017) OR LIMIT-TO ( PUBYEAR,2016) OR LIMIT-TO ( PUBYEAR,2015) OR LIMIT-TO ( PUBYEAR,2014) OR LIMIT-TO ( PUBYEAR,2013) OR LIMIT-TO ( PUBYEAR,2012) OR LIMIT-TO ( PUBYEAR,2011) OR LIMIT-TO ( PUBYEAR,2010) OR LIMIT-TO ( PUBYEAR,2009) )

### Web of Science

TS=(cerebral palsy) AND TS= (play* OR game OR gamification OR toy) AND TS=(therapy OR rehabilitation OR treatment) AND TS=(child*) AND TS= (hand OR "upper limbs")

**YEARS:** ( 2022 OR 2021 OR 2020 2019 OR 2014 OR 2009 OR 2018 OR 2013 OR 2017 OR 2012 OR 2016 OR 2011 OR 2015 OR 2010 )

### CINAHL

(”cerebral+palsy”+OR+”cerebral+paresis”+OR+”cerebral+palsies”+)+AND+(+play*+OR+game+OR+gamification+OR+toy+)+AND+child*+AND+(+therapy+OR+rehabilitation+OR+treatment+)+AND+(+hand+OR+”upper+limbs”+)&DT1=200901-202212
