# Supplementary material for: Playfulness and New Technologies in Hand Therapy for Children With Cerebral Palsy: Scoping Review
Source: JMIR Serious Games. 2023 Oct 16;11:e44904. doi: 10.2196/44904 (PMC10616756; doi:10.2196/44904)
Supplement: Multimedia Appendix 3 [file games_v11i1e44904_app3.doc]

## Multimedia Appendix 3 – Types of hardware

| **Type of hardware** | **Name** | **Amount** |
| --- | --- | --- |
| Computers  n=32 | PC | (n=26) [31] [33][35] [36][37][42][44][46][47] [49][50] [51] [52][53][54][55] [59][60][61] [64][69][71][72][73] [75][76] |
|  | Laptop | (n=4) [65][66][68][70] |
|  | Tablet | (n=2) [67][74] |
| Controllers  n=11 | Custom levers | (n=3) [65][70][75] |
|  | Geomagic Touch | (n=1) [52] |
|  | Joystick | (n=2) [57][76] |
|  | Motion Therapy mouse | (n=1) [69] |
|  | Novint Falcon | (n=3) [35] [42][65] |
|  | Wii Nunchuk | (n=1) [41] |
| Game consoles  n=11 | Nintendo Wii | (n=6) [29][30][32][34][45][9] |
|  | Play Station | (n=4) [38][39][40][48] |
|  | XBox | (n=1) [56] |
| Motion Sensing  n=14 | EyeToy | (n=1) [48] |
|  | HTC Vive trackers | (n=1) [51] |
|  | IREX | (n=1) [25] |
|  | Kinect | (n=6) [31] [36] [44][46][47][56] |
|  | Leap Motion | (n=5) [30][33] [37][54][55] |
| Robotics  n=2 | Robotic Arm | (n=2) [60][71] |
| Smart Tangibles  n=18 | Interactive Story box | (n=1) [28] |
|  | Lego Mindstorms NXT | (n=1) [43] |
|  | Multitouch display | (n=2) [47][63] |
|  | Pleo! | (n=1) [41] |
|  | PhysiTable | (n=1) [26] |
|  | Ride-on-toy | (n=1) [57] |
|  | Smart blocks | (n=1) [24] |
|  | Smart toys | (n=8) [27][61] [62] [66] [72] |
|  | TagTiles | (n=1) [27] |
|  | Tangible objects | (n=1) [63] |
| VR headsets  n=2 | Oculus Rift | (n=2) [52,58] |
| Wearables  n=19 | 5DT sensing gloves | (n=2) [38] [39] |
|  | Accelerometer | (n=1) [63] |
|  | Arm and elbow remote | (n=2) [61][62] |
|  | Arm sensors | (n=4) [58] [64] [68] [72] |
|  | ﻿Electrical stimulation electrodes | (n=1) [64] |
|  | Data glove | (n=1) [73] |
|  | Mindwave | (n=1) [33] |
|  | Music glove | (n=1) [74] |
|  | Myo armband | (n=2) [49][50] |
|  | Neofect Smart Kids | (n=2) [59] [67] |
|  | Polhemus Liberty | (n=1) [53] |
