# Supplementary material for: Playfulness and New Technologies in Hand Therapy for Children With Cerebral Palsy: Scoping Review
Source: JMIR Serious Games. 2023 Oct 16;11:e44904. doi: 10.2196/44904 (PMC10616756; doi:10.2196/44904)
Supplement: Multimedia Appendix 4 [file games_v11i1e44904_app4.doc]

## Multimedia Appendix 4 –Studies that use a combination of different types of hardware.

| **Combined Technologies** | **Study** |
| --- | --- |
| Arm sensors and Laptop | [68] |
| Arm sensors and Oculus Rift | [58] |
| Arm Sensors, electrical stimulation electrodes, and PC | [64] |
| Custom lever and Laptop | [70] |
| Custom levers and PC | [75] |
| Custom levers, Laptop and Novint Falcon | [65] |
| Data glove and PC | [73] |
| Geomagic Touch, Oculus Rift and PC | [52] |
| HTC Vive trackers and PC | [51] |
| Joystick and PC | [76] |
| Kinect and PC | [31] [36] [44][46] |
| Kinect, Multitouch display and PC | [47] |
| Leap Motion and Nintendo Wii | [30] |
| Leap Motion and PC | [37][54][55] |
| Leap Motion, Mindwave and PC | [33] |
| Motion (therapy) mouse | [69] |
| Music glove and Tablet | [74] |
| Myo armband and PC | [49][50] |
| Neofect Smart Kids and PC | [59] |
| Neofect Smart Kids and Tablet | [67] |
| Novint Falcon and PC | [35,42] |
| Play Station and EyeToy | [48] |
| Play Station and 5DT sensing gloves | [38,39] |
| Polhemus Liberty and PC | [53] |
| Ride-on-Toy and Joystic | [57] |
| Robotic Arm and PC | [60] [71] |
| Smart Toy and Arm, elbow remote | [62] |
| Smart Toys and Arm, elbow remote and PC | [61] |
| Smart toys and Laptop | [66] |
| Smart Toys, arm sensors and PC | [72] |
| TagTiles and SmartToys | [27] |
| Tangible objects, accelerometer, and Multitouch Display | [63] |
| Wii Nunchuk and Pleo | [41] |
| Xbox and Kinect | [56] |
